# Physiological Perspectives on the Use of Triheptanoin as Anaplerotic Therapy for Long Chain Fatty Acid Oxidation Disorders

**DOI:** 10.3389/fgene.2020.598760

**Published:** 2021-01-15

**Authors:** Evgenia Sklirou, Ahmad N. Alodaib, Steven F. Dobrowolski, Al-Walid A. Mohsen, Jerry Vockley

**Affiliations:** ^1^Department of Pediatrics, School of Medicine, University of Pittsburgh, Pittsburgh, PA, United States; ^2^Newborn Screening and Biochemical Genetics Lab, Department of Genetics, King Faisal Specialist Hospital & Research Centre, Riyadh, Saudi Arabia; ^3^Department of Pathology, School of Medicine, University of Pittsburgh, Pittsburgh, PA, United States; ^4^Department of Human Genetics, Graduate School of Public Health, University of Pittsburgh, Pittsburgh, PA, United States; ^5^Center for Rare Disease Therapy, UPMC Children’s Hospital of Pittsburgh, Pittsburgh, PA, United States

**Keywords:** metabolomics, very long chain acyl-CoA dehydrogenase, fatty acid oxidation disorders, energy metabolism, tricarboxylic acid cycle, anaplerosis, inborn errors of metabolism, fatty acid oxidation

## Abstract

Inborn errors of mitochondrial fatty acid oxidation (FAO) comprise the most common group of disorders identified through expanded newborn screening mandated in all 50 states in the United States, affecting 1:10,000 newborns. While some of the morbidity in FAO disorders (FAODs) can be reduced if identified through screening, a significant gap remains between the ability to diagnose these disorders and the ability to treat them. At least 25 enzymes and specific transport proteins are responsible for carrying out the steps of mitochondrial fatty acid metabolism, with at least 22 associated genetic disorders. Common symptoms in long chain FAODs (LC-FAODs) in the first week of life include cardiac arrhythmias, hypoglycemia, and sudden death. Symptoms later in infancy and early childhood may relate to the liver or cardiac or skeletal muscle dysfunction, and include fasting or stress-related hypoketotic hypoglycemia or Reye-like syndrome, conduction abnormalities, arrhythmias, dilated or hypertrophic cardiomyopathy, and muscle weakness or fasting- and exercise-induced rhabdomyolysis. In adolescent or adult-onset disease, muscular symptoms, including rhabdomyolysis, and cardiomyopathy predominate. Unfortunately, progress in developing better therapeutic strategies has been slow and incremental. Supplementation with medium chain triglyceride (MCT; most often a mixture of C8–12 fatty acids containing triglycerides) oil provides a fat source that can be utilized by patients with long chain defects, but does not eliminate symptoms. Three mitochondrial metabolic pathways are required for efficient energy production in eukaryotic cells: oxidative phosphorylation (OXPHOS), FAO, and the tricarboxylic (TCA) cycle, also called the Krebs cycle. Cell and mouse studies have identified a deficiency in TCA cycle intermediates in LC-FAODs, thought to be due to a depletion of odd chain carbon compounds in patients treated with a predominantly MCT fat source. Triheptanoin (triheptanoyl glycerol; UX007, Ultragenyx Pharmaceuticals) is chemically composed of three heptanoate (seven carbon fatty acid) molecules linked to glycerol through ester bonds that has the potential to replete TCA cycle intermediates through production of both acetyl-CoA and propionyl-CoA through medium chain FAO. Compassionate use, retrospective, and recently completed prospective studies demonstrate significant reduction of hypoglycemic events and improved cardiac function in LC-FAOD patients, but a less dramatic effect on muscle symptoms.

## Introduction

Long-chain fatty acid oxidation disorders (LC-FAODs) are a rare group of inborn errors of metabolism that affect several enzymes involved in the pathway of mitochondrial β-oxidation, including both the transportation of long-chain fatty acids into the mitochondria through the carnitine shuttle, as well as the process of chain shortening of acyl-CoA substrates (Knottnerus et al., 2018). Fatty acid oxidation (FAO) ultimately leads to the production of energy during times of fasting and physiologic stress. In addition to an energy deficit that impacts multiple organ systems, the incomplete oxidation of fatty acids also causes accumulation of toxic long-chain acyl-CoA intermediates. The disorders of the LC-FAODs are autosomal recessive and include carnitine palmitoyl transferase 1 (CPTI), carnitine palmitoyl transferase 2 (CPTII), carnitine-acylcarnitine translocase (CACT), very long-chain acyl-CoA dehydrogenase (VLCAD), long-chain 3-hydroxyacyl-CoA dehydrogenase (LCHAD), and mitochondrial trifunctional protein (TFP) deficiencies (Knottnerus et al., 2018).

Epidemiologic studies of NBS populations indicate that the combined incidence of all FAODs ranges from 0.9–15.2 per 100,000 (Marsden et al., 2020). Very long-chain acyl-CoA dehydrogenase (VLCAD) deficiency is the most prevalent LC-FAOD in most populations, with incidences ranging from 0.07–1.9 per 100,000, whereas other LC-FAODs have a low incidence (<1.0 per 100,000) (Marsden et al., 2020).

LC-FAODs present with variable clinical manifestations and severity ranging from early neonatal presentations with hypoketotic hypoglycemia, metabolic acidosis, cardiomyopathy, severe hepatopathy, multiorgan failure and death, to later onset disease presentations with recurrent episodes of rhabdomyolysis, exercise intolerance, myopathy, peripheral neuropathy and retinopathy. Cardiomyopathy and cardiac arrhythmias can occur at any age (Baruteau et al., 2013). Patients are at constant risk for a metabolic decompensation during periods of increased energy requirements such as prolonged fasting or illness. LC-FAODs are currently included in the newborn screening programs in many countries including the US, allowing early intervention to prevent the devastating early manifestations as well as the chronic disease-related complications. However, despite early diagnosis and appropriate treatment, most patients still experience disease-related complications and long-term disabilities (Spiekerkoetter et al., 2009a).

Medical treatment for LC-FAODs is consensus and experience driven rather than evidence based (Vockley et al., 2002; Spiekerkoetter et al., 2009b; Knottnerus et al., 2018). The cornerstone of management is avoidance of fasting in order to reduce the need of fatty acid oxidation. Treatment often includes a diet that is rich in carbohydrates and low in long-chain fats and is usually supplemented with medium even chain triglycerides (MCT oil), as well as essential fatty acids to prevent possible deficiencies secondary to dietary fat restriction (Spiekerkoetter et al., 2009b; Spiekerkoetter and Mayatepek, 2010; Knottnerus et al., 2018). The use of L-carnitine is controversial due to the concern about the potential arrhythmogenic potential of long-chain acylcarnitines, but it is sometimes prescribed if blood free carnitine levels are felt to be too low. Emergency protocols with intravenous glucose supplementation must be promptly initiated in acute clinical presentations with sickness and inability to maintain adequate oral intake. However, progress in treating LC-FAODs has been achieved with the introduction of trihepatnoin, an odd medium chain fatty acid in a triglyceride form (triheptanoyl glycerol).

## Mitochondrial Fatty Acid Oxidation

**FIGURE 1:**
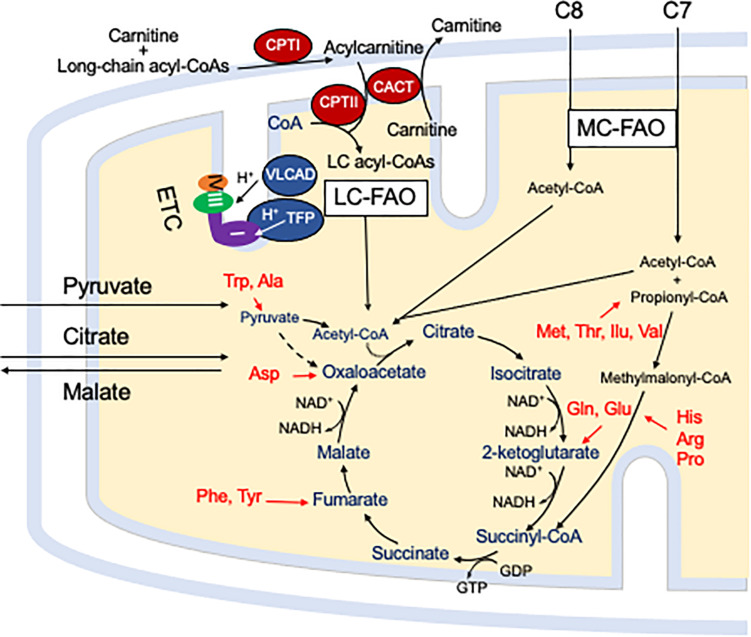
The carnitine cycle involves generation of a long chain acylcarnitine from activated fatty acids by CPT I, transport across the mitochondrial membranes by CACT, and release of the activated acyl-CoA substrate for FAO into the mitochondrial matrix by CPT II. Carnitine itself is transported into cells by a high affinity transporter (OCTN2). All abbreviations are as defined in the text. Interactions of mitochondrial energy metabolism pathways. The carnitine cycle transport long-chain CoAs into the mitochondrial matrix with carnitine ultimately being restored to the cytoplasm in exchange for release of an acyl-CoA in the mitochondrial matrix. Medium chain fats directly diffuse into mitochondria. ETC and the enzymes of LC-FAO functionally and physically interact. Mitochondrial TFP interacts with and passes its reducing equivalents to the matrix arm of ETC complex I. VLCAD interacts with TFP, but its reducing equivalents are transferred to ETC complex III via electron transfer flavoprotein (ETF, not shown) and ETF dehydrogenase (not shown). The acetyl-CoA product of one cycle of FAO enters the TCA cycle to produce citrate. While standard medium chain trioctanoylglycerol (C8) provides double the amount of acetyl-CoA compared to triheptanoin (C7), the latter provides a propionyl-CoA that enters the TCA cycle through succinyl-CoA. Anaplerotic amino acids are listed in red. Pyruvate and citrate enter the mitochondria through specific carriers (the latter with counter transport of malate to the cytoplasm). All abbreviations are as defined in the text.

**FIGURE 2:**
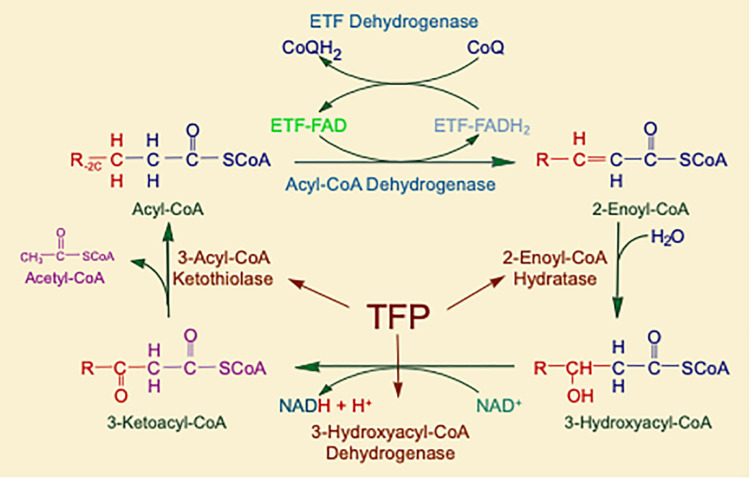
The matrix reactions of FAO consist of four steps that result in one molecule of acetyl-CoA and an acyl-CoA that is shortened by two-carbon units. For long-chain substrates, VLCAD, which catalyzes the first step is long-chain fatty acid β-oxidation, is the most important acyl-CoA dehydrogenase for energy generation. The next three steps are performed by a single multifunctional enzyme with three active sites, the mitochondrial TFP. All abbreviations are as defined in the text.

Mitochondrial fatty acid oxidation (FAO) is the process by which fatty acids from endogenous stores (usually 16 or 18 carbons in chain length) or, in some situations, from the diet are metabolized for energy (Vockley et al., 2016a). Typically during times of metabolic stress or physiologic fasting, endogenous fatty acids, typically stored in the body as long-chain triglycerides, are mobilized through the action of lipases, transported through the blood stream to cells by carrier proteins, then taken up by peripheral cells through specific membrane receptors/transporters where they are activated to acyl-CoAs by a set of acyltransferases. Medium and short chain acyl-CoAs can enter mitochondria directly, but substrates longer than 10 carbons use a series of reactions known as the carnitine cycle for transport across the mitochondrial membrane (Figure 1) (Longo, 2016). Here, the acyl-CoA is first conjugated to carnitine by carnitinepalmitoyl transferase 1 (CPTI), shuttled across the intermembrane space by carnitine-acylcarnitine translocase (CACT) in exchange for carnitine, then the acyl-CoA is released in the mitochondrial matrix along with free carnitine by carnitinepalmitoyl transferase 2 (CPTII). In the mitochondrial matrix, a series of four enzymatic reactions cleaves the two carbon moiety acetyl-CoA and leaves an acyl-CoA with a primary carbon backbone that is two carbons shorter (Figure 2) (Vockley et al., 2016a). These reactions are catalyzed by families of chain length optimized enzymes. In the first, the acyl-CoA is oxidized to an enoyl-CoA by the acyl-CoA dehydrogenases (ACADs), including enzymes with substrate optima of 16, 8, and 4 carbons in chain length (very long-, medium- and short chain ACADs; VLCAD, MCAD, and SCAD, respectively) (Swigonova et al., 2009). The next three steps for long-chain acyl-CoAs [enoyl-CoA hydratase (ECDH), long-chain 3-hydroxyacyl-CoA dehydrogenase (LCHAD), and 3-ketoacyl-CoA thiolase (KAT)] are catalyzed by a single multimeric enzyme known as the mitochondrial trifunctional protein (TFP), while individual enzymes specific to each reaction exist for medium and short chain substrates (Liang et al., 2018; Xia et al., 2019). Additional enzymes are required to completely metabolize unsaturated fats. The acetyl-CoA final product of FAO is available to participate in other mitochondrial process [most often the tricarboxylic acid (TCA) or Krebs cycle] or in liver can enter in the ketone synthesis pathway to make 3-hydroxybutyrate and acetoacetate, alternative fuels that can be used by many tissues.

## Interaction of Mitochondrial Energy Pathways

Energy generation in the mitochondria occurs through the intersection of three major enzyme pathway, oxidative phosphorylation (OXPHOS), FAO and the TCA cycle (Figure 1) (Vockley et al., 2019b). Reducing equivalents are generated at two steps in FAO, by ACADs with production of a reduced FAD cofactors, and by 3-hydroxyacyl-CoA dehydrogenase, utilizing nicotin adenine dinucleotide (NAD) as the electron acceptor. The ACADs are re-oxidized by electron transfer flavoprotein (ETF), which in turn is re-oxidized by the ETF dehydrogenase (ETFDH, also known as ETF-Coenzyme Q oxidoreductase). Finally, reducing equivalents are passed from ETFDH to complex III of the electron transfer chain (ETC). Electrons from reduced NADH (NADH) are direct substrates for complex I of the ETC. The enzymes of long-chain FAO and the ETC are both functionally and physically associated in a macromolecular energy complex. The LCHAD subunit of TFP interacts with the matrix arm of complex I, while ETFDH interacts with the cor2 subunit of complex III. VLCAD binds with the ECDH subunit of TFP, thus creating a metabolon that optimizes enzymatic efficiency of the linked enzymatic processes.

## Disorders of Fatty Acid Oxidation

Inborn errors of LC-FAOD are among the most common in humans, affecting ∼1:10,000 babies born. At least 25 enzymes and specific transport proteins are responsible for carrying out the steps of mitochondrial fatty acid metabolism, with at least 22 associated genetic disorders (Wilcken et al., 2009; Lindner et al., 2010). Most patients with LC-FAODs in the US are now identified pre-symptomatically through newborn screening. Common symptoms in the first week of life include cardiac arrhythmias, hypoglycemia, and sudden death (Knottnerus et al., 2018). Symptoms later in infancy and early childhood may relate to liver, cardiac, or skeletal muscle dysfunction, and include fasting or stress-related hypoketotic hypoglycemia or Reye-like syndrome, cardiomyopathy, and recurrent rhabdomyolysis. In adolescents or adults, muscular symptoms, including rhabdomyolysis and cardiomyopathy predominate. Progressive peripheral neuropathy and retinopathy are unique to TFP/LCHAD deficiency (Spiekerkoetter et al., 2004; Rector et al., 2008). Current therapies do not address the basic pathologies of LC-FAODs (Spiekerkoetter and Mayatepek, 2010). MCT oil provides a medium chain fat source (octanoate in its most purified form) that can be utilized by patients with long-chain FAO defects, but does not eliminate symptoms. Mutations have been described in numerous patients with LC-FAODs, but genotype-phenotype correlations are imperfect (Nahabet et al., 2016).

### TCA Cycle Depletion and the Role of Anaplerosis in Treating FAODs

In the early years after the discovery of the LC-FAODs, especially VLCAD and TFP/LCHAD deficiencies, the expectation of most metabolic specialists was that MCT oil should completely rescue patients from the effects of these disorders because of an ample supply of acetyl-CoA groups to the TCA cycle. While it was at least partially effective, some symptoms persisted and, in many cases, worsened again with time. This observation led to the novel suggestion that loss of some TCA cycle intermediates through either cellular damage or use in other metabolic pathways led to a depletion of those substrates generated from odd chain carbon sources (i.e., succinate from propionyl-CoA) that led to energy impairment regardless of continued delivery of acetyl-CoA from octanoate. Triheptanoin, the triglyceride of heptanoate, was proposed as an alternative treatment that could directly address both TCA cycle substrate needs in LC-FAODs since it still bypassed the need for long-chain fat transport and catabolism and was metabolized to two molecules of acetyl-CoA and one of propionyl-CoA. Indeed, the first patient treated with this compound, a 5 years old with VLCAD deficiency and persistent cardiomyopathy, responded to the medication with dramatic improvement of heart function (see below) (Roe et al., 2002). Subsequent metabolic flux experiments in rats demonstrated that intra-duodenal infusion of heptanoate led to nearly complete uptake in the liver and stimulated production of C5 ketone bodies presumed to be synthesized from propionyl-CoA (Deng et al., 2009). However, it was subsequently demonstrated that nearly all of the liver generated propionyl-CoA was channeled to the TCA cycle, indicating that the C5-ketone bodies were being derived instead from the partial heptanoate degradation to produce valeryl-CoA that was then used for odd chain ketone body synthesis (Kinman et al., 2006). In addition, the incorporation of label from heptanoate into C5-ketone bodies was lower than that from labeled octanoate into C4-ketone bodes. Thus, the anaplerotic potential of heptanoate is greater than that of octanoate. Original concern that stimulation of lipolysis of long-chain lipids by intravenous infusion of heptanoate might be limited in this setting were negated by additional studies showing that they were in fact efficiently re-esterified with no effect on delivery of long-chain fats into FA (Kinman et al., 2006; Gu et al., 2010). Induction of ketosis through a ketogenic diet for 60 days with or without triheptanoin showed that hexadecanoic, 9-hexadecenoic, and 9-octadecenoic were elevated in the livers of the triheptanoin fed animals (Vieira de Melo et al., 2015). The physiologic implications of these findings are unclear as both sets of animals appeared well. An MCT arm was not included in the study.

Additional information on the disposition of ingested triheptanoin has been obtained in long term studies on mice. After 1 year of supplementation with either C8 MCT oil or triheptanoin, both wild type and VLCAD deficient mice demonstrated a significant accumulation of a variety of even and odd chain complex lipids, respectively, in liver including heptadecenoic acid (C17:1n9), eicosanoic acid (C20:1n9), erucicacid (C22:1n9), and mead acid (C20:3n9) (Tucci et al., 2015). Peroxisomal fatty acid oxidation was also increased in these animals compared to normal diet, consistent with increased flux through complex lipid metabolic pathways. However, only animals fed C8 had upregulation of the peroxisomal L-bifunctional fatty acid oxidase protein, a finding associated with increased toxicity presumed to be related to increased production of reactive oxygen species. Heart lipid composition was also altered with an increase in monounsaturated fats in C8 and C7 fed mice and a corresponding decrease in polyunsaturated fats. Elongation of the medium chain fats in this model was supported by an upregulation of the sterol element binding transcription factor 1 that regulates many of the genes involved in lipid synthesis as well as increased expression of several genes in lipid chain length elongation. Finally, VLCAD deficient mice show an age dependent shift in muscle fibers from oxidative to the glycolytic type. This shift is inhibited when mice are treated with MCT oil, but not with triheptanoin. In total, these studies indicate that both MCT (C8) and triheptanoin induce changes in the lipidome with an increase in long-chain fats due to elongation of the octanoyl- and heptanoyl-CoA substrates. However, the former seems to have more potential for toxicity than the latter.

### Metabolomic Abnormalities in VLCAD Deficient Patients

Given these animal findings, we sought to explore changes that might be occurring in patients with VLCAD deficiency. Because of participation in clinical trials for FDA approval of triheptanoin, we had a large biobank of samples from patients on standard of care (MCT oil) and triheptanoin. We selected plasma samples from 8 patients each on MCT oil or on triheptanoin and sent them to Metabolon^1^ for evaluation of small molecule metabolomics using their clinically validated MetaGA platform (Dobrowolski et al., 2020). Briefly, this test applies three platforms for analysis: liquid chromatography/tandem mass spectrometry (LC-MS/MS) optimized for basic species, LC-MS/MS optimized for acidic species, and gas chromatography/mass spectrometry (Evans et al., 2009; Dehaven et al., 2010). Sample preparation for gas chromatography/mass spectrometry utilized bistrimethyl-silyl-trifluoroacetamide derivatization in a acetonitrile:dichloromethane:cyclohexane (5:4:1) solvent. LC-MS/MS utilized a Waters Acquity UPLC (Waters, Millford, MA, United States) and an LTQ mass spectrometer (Thermo Fisher Scientific, Inc., Waltham, MA, United States) assessing masses of 99–1,000 m/z as described. Gas chromatography/mass spectrometry applied a 5% phenyldimethyl silicone column (60–340°C) with a Thermo-Finnigan Trace DSQ MS (Thermo Fisher Scientific, Inc.) to assess masses of 50–750 atomic mass units as described. Data analysis was performed with company proprietary software as previously described (Quell et al., 2017). Briefly, automated and visual comparison of ion features to reference standards (i.e., retention time, mass-to-charge ratio, fragmentation mass spectra) was determined. Statistical analysis was performed using “R” version 2.14. Welch’s *t*-tests compared data from patient blood to a validated age and sex match normal control database. The calculated *z*-scores show deviation from the mean of age matched controls measured and curated by the clinical testing lab. Analyte differences were considered significantly with *p* ≤ 0.05. Thus, all patient samples are compared to normal population controls rather than each other.

**FIGURE 3:**
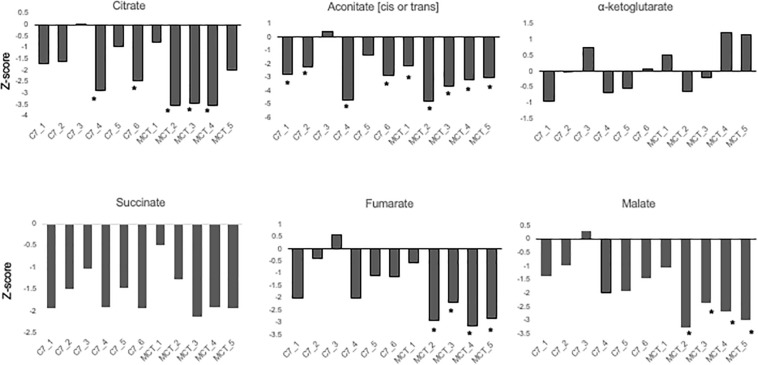
Concentrations of TCA cycle intermediates in the blood from VLCAD deficiency patients treated with standard MCT oil (MCT1–5) or triheptanoin (C1–6). The values are given as *Z*-score, defined as the number of standard deviations from age and sex matched control means.

**FIGURE 4:**
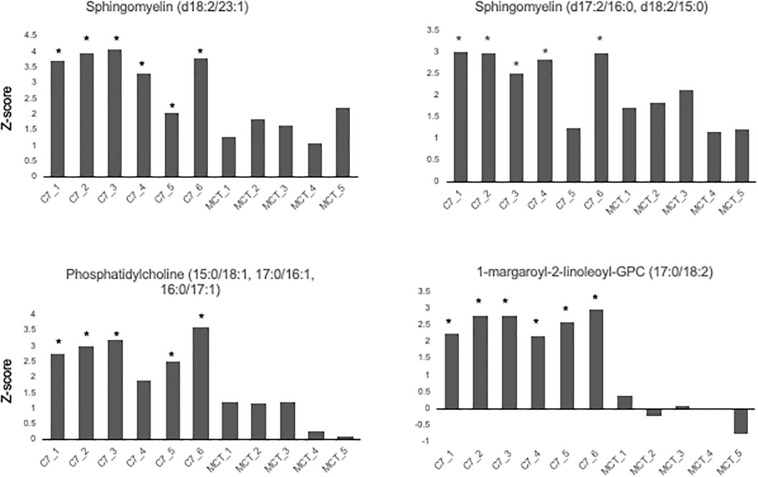
Concentrations of odd chain long-chain complex lipids in VLCAD patients treated with MCG oil (MCT 1–5) or triheptanoin (C7 1–6). The values are given as *Z*-score, defined as the number of standard deviations from age and sex matched control means. Compound abbreviation are as defined in Table 1.

Results are shown in Table 1 and Figures 3, 4. With the exception of α-ketoglutarate, representation of Krebs’ cycle intermediates trend lower in VLCAD deficient patients (Figure 3). However, citrate, aconitate, fumarate and malate are higher in those treated with C7. Reduced succinate is common to both treatment groups suggesting rapid utilization of this metabolite. These findings support the anaplerotic role of C7 in supplementation of the Krebs’ cycle via combined propionyl-CoA and acetyl-CoA production. Analysis of patients revealed enrichment of very long-chain carnitine esters including arachidoylcarnitine (C20), behenoylcarnitine (C22), and nervonoylcarnitine (C24:1) regardless of treatment (Table 1 and Figure 4). Patients treated with C7 also showed an increase of unique odd chain sphingomyelins, phosphatidylcholines, and phosphatidylethanolamines compared to control and patients treated with MCT. While the mechanism for the increase and pattern of these metabolites remains to be proven, studies in VLCAD deficient mice suggest that that they are due to excess substrate of the supplemented medium chain oil (C7 or C8) that is then chain elongated and participates in increased complex lipid synthesis (Tucci et al., 2015; Tucci, 2017; Tucci et al., 2017). Regardless of mechanism, these results demonstrate that long-chain complex lipids with significant signaling potential to induce inflammation accumulate in VLCAD deficient patients, regardless of treatment with C7 and C8. These findings raise the possibility that while a bioenergetic deficit plays a major role in the development of hypoglycemia and cardiomyopathy, the accumulation of abnormal long-chain fats serve as a signal for inflammation and subsequent rhabdomyolysis.

**TABLE 1:** Complex lipids significantly increased in VLCAD patients.

Pathway	Metabolite
Lipid synthesis	
	Arachidoylcarnitine (C20)
	Margaroylcarnitine (C17)
	Nervonoylcarnitine (C24:1)
Lysophospholipid	
	1-margaroyl-glycerophosphoethanolamine (C17)
	1-pentadecanoyl- glycerylphosphorylcholine (C15)
Phosphatidylcholine	
	1-margaroyl-2-arachidonoyl-glycerylphosphorylcholine (17:0/20:4)
	1-margaroyl-2-docosahexaenoyl-glycerylphosphorylcholine (17:0/22:6)
	1-margaroyl-2-linoleoyl- glycerylphosphorylcholine (17:0/18:2)
	1-pentadecanoyl-2-arachidonoyl- glycerylphosphorylcholine (15:0/20:4)
	1-pentadecanoyl-2-docosahexaenoyl- glycerylphosphorylcholine (15:0/22:6)
	Phosphatidylcholine (15:0/18:1, 17:0/16:1, 16:0/17:1)
Sphingolipid	
	Sphingomyelin (d18:2/23:1)
	Sphingomyelin (d17:2/16:0, d18:2/15:0)
Amino acid	
	Gamma-glutamylglycine

## Treatment of LC-FAODs With Triheptanoin

Until recently, no medications had been approved for treatment of long-chain fatty acid oxidation disorders. However, the U.S. Food and Drug Administration (FDA) approved Dojolvi (generic name triheptanoin, also known as UX007 when in clinical trials), in June 2020, for the treatment of pediatric and adult patients with LC-FAODs. The use of triheptanoin in treatment of LC-FAODs is based on its action as an anaplerotic molecule that can correct secondary depletion of TCA cycle intermediates that occurs in these disorders (Roe et al., 2002). Table 2 summarizes published studies on the use of triheptanoin in LC-FAODs. The first report was three VLCAD deficient patients with early presentation including neonatal hypoglycemia, cardiomyopathy in infancy, muscle weakness, and subsequent recurrent episodes of rhabdomyolysis. At enrollment, one patient had persistent cardiomyopathy at 5 years old, and two had hepatomegaly prior to triheptanoin initiation at 2 and 9 years old, respectively. Following triheptanoin, all patients showed marked improvement of strength, endurance and activity, the cardiomyopathy improved in the 5 years old, and the hepatomegaly resolved in the other two patients. The family of the 2 years old patient elected to discontinue the triheptanoin diet and returned to previously administered MCT diet, with subsequent recurrence of rhabdomyolysis episodes. Both four-carbon ketone bodies (acetoacetate, β-hydroxybutyrate) and five-carbon ketones (β-hydroxypentanoate, β-ketopentanoate) were substantially increased in triheptanoin treated patients, while disease specific markers (C14:1) were reduced and C3 was increased.

**TABLE 2:** Triheptanoin studies for LC-FAODs.

Study (References)	Design	Sample size	Disease	Clinical presentation	Intervention	Outcomes
Roe et al., 2002; Mochel et al., 2005	Case series	3 (age 2,6 9 y)	VLCAD	Neonatal hypoglycemia (3 pt), cardiomyopathy (1 pt), muscle weakness (3 pt), hepatomegaly (2 pt), recurrent rhabdomyolysis episodes (2 pt).	Triheptanoin 2.6–4 gr/kg/day	Cardiomyopathy improved, hepatomegaly resolved, improved muscle strength, improved endurance and activity. One pt discontinued C7 and rhabdo episodes recurred.
Roe et al., 2008; Breen et al., 2014	Case series	7 (10–55 y)	CPTII	6 pts hospitalized at least once for rhabdo episodes. Hx of exercise restriction due to muscle pain	Triheptanoin 1-2 gr/kg/day (adolescents, adults). Triheptanoin 3–4 gr/kg/day (for pts <12 y), div QID. Prior to C7, 41 pts on MCT	Improvement in daily activities. None hospitalized for rhabdo while on C7. All pts able to compete in team sports. 2 pts had mild muscle pain w exercise. 2 pts admitted with rhabdo with strenuous exercise after discontinuing C7 × 1–2 weeks.
Pascual et al., 2014; Roe and Brunengraber, 2015	Case series	52 (birth-51 y)	CACT (2) CPTI (2) CPTII (11) LCHAD (10) TFP (6) VLCAD (21)	3 pts started C7 < 1 m old (1 at birth). 1 infant with CACT with ventricular arrhythmia, cardiomyopathy, hyperammonemia, hepatomegaly, hypotonia. 2 pregnant pts with VLCAD/rhabdo during previous pregnancies, muscle pain, weakness.	C7 at 30–35% of total dailt calories; 1 gr/kg/day (adolescents, adults), 3–4 gr/kg/day (<12 y)	SCC reduced (∼60% > ∼10%). Mortality decreased (∼65% > ∼3.8%). Rhabdo reduced (85% > 31%). Weakness decreased (92% > 12%). Hypoglycemia not reported (42% on previous conventional diet). Hepatomegaly, elev LFTs reduced (46% > 6%) Cardiomyopathy: 1 pt with VLCAD on C7, 18/52 pts before C7
Vockley et al., 2015; Mochel et al., 2016	Chart review, Retrospective	20	CACT (1) CPTII (3) LCHAD (5) TFP (2) VLCAD (9)	Cardiomyopathy Rhabdomyolysis Muscle pain Hypoglycemia	Analyzed pts’ clinical course for the 2 years before and after the start of C7. 85% pt on MCT before.	35% reduction in mean hospitalizations/year. 67% decrease in mean total hospital days/year. Hypoglycemia event rate: 96% decrease. Cardiomyopathy improved (3/6), stable (1/6). Rhabdo event rate not change substantially (however, 60% decrease in mean hospital days/year)
Vockley et al., 2016b; Hainque et al., 2019	Case series	10 (2.5 m–20 y) 8pts < 1 y	CACT (2) LCHAD (2) TFP (2) VLCAD (4)	Cardiomyopathy. 9/10pts presented in infancy and most required ECMO and/or vasopressors	C7 at 25–35% of total calories	EF improvement between 2 and 21 ds. Mean EF increased to 60% (range 33–71) ECMO and/or pressors discontinued in 2–10 days 2 deaths: 3.5 m with VLCAD died of sepsis. 3.5 m old with TFP died of refractory cardiogenic shock.
Gillingham et al., 2017; Hainque et al., 2020	Double blind, randomized controlled trial	32	CPTII (11) LCHAD/TFP (12) VLCAD (9)	Recurrent episodes of rhabdomyolysis 1 pt with VLCAD with abnormal cardiac function. 1 pt with LCHAD had cardiac arrest with resuscitation.	Pts received diet with C7 vs. C8 at 20% of their total daily calories × 4 m	C7 pts: increased LVEF by 7.4%. C7 pts: exercise at lower max heart rate. Muscle symptoms: no difference between C7, C8 groups. Rhabdo admissions: Same in both groups. Admission length: Same in both groups.
Roe et al., 2010; Vockley et al., 2017	Single arm Open label	29 (10 m–58 y)	CPTII (4) LCHAD (10) VLCAD (12) TFP (3)	Skeletal myopathy^*a*^ Hypoglycemia Cardiac disease. Hepatic disease.	C7 at 25–35% of total daily caloric intake × 24 weeks	12-MWT: 28% increase from baseline. ETT: 60% increase in generated watts. HR-QoL: significant improvement in 5/6 adults. No change from baseline in children.
Calvert et al., 2018; Vockley et al., 2019a	Single arm. Open label.	29 (10 m–58 y)	CPTII (4) LCHAD (10) VLCAD (12) TFP (3)	Skeletal myopathy^*a*^. Hypoglycemia Cardiac disease. Hepatic disease.	C7 at 25–35% of total daily caloric intake × 78 weeks	MAMER: 48% decrease. MAMEDR: 50.3% decrease. MAHR: 53.1% decrease. MAHDR: 51.5% decrease. MARR: 36.1% decrease. MARDR: 29.3% decrease. Mean annualized hypoglycemia event rate: 92.8% decrease.

*abn, abnormal; CACT, carnitine-acylcarnitine translocase; CPTI, carnitine palmitoyltransferase I; CPTII, carnitine palmitoyltransferase II; EF, ejection fraction; ETT, exercise tolerance test; HR-QoL, health related quality of life; LCHAD, long-chain 3-hydroxyacyl-CoA dehydrogenase; LFTs, liver function tests; LVEF, left ventricular ejection fraction; m, month(s); max, maximum; MAHDR, mean annualized hospitalization duration rate; MAHR, mean annualized hospitalization rate; MAMEDR, mean annualized major event duration rate; MAMER, mean annualized major event rate; MARDR, mean annualized rhabdomyolysis duration rate; MARR, mean annualized rhabdomyolysis rate; SCC, serious clinical complications; TFP, trifunctional protein; w, with; wk, week(s); y, year; 12MWT, 12-min walk test.^*a*^Rhabdomyolysis, muscle pain, exercise intolerance, muscle weakness.*

Triheptanoin was also studied in 7 patients with CPTII deficiency with an age range 10–55 years’ old for 7–61 months (Roe et al., 2008). All patients but one, had been hospitalized at least once for episodes of rhabdomyolysis and had been restricting exercise due to muscle pain on exertion. Their previous diet management consisted of low fat and increased carbohydrates, while patients younger than 14 years also received MCT oil comprising 6–10% of their daily caloric intake. By the end of the first week, most patients experienced some improvement in daily activities and within 1–2 months, their exercise tolerance was enhanced. While on triheptanoin, none was admitted due to rhabdomyolysis and two experienced mild muscle pain with exercise. All participants were able to compete in sports, a previously restricted activity. Two patients presented with rhabdomyolysis following strenuous exercise requiring admission after discontinuing triheptanoin use for 1–2 weeks.

A large retrospective review of 52 patients treated with triheptanoin included 8 infants, 28 children, 7 adolescents, and 9 adults with CPT-I, CACT, CPT II, VLCAD, TFP, and LCHAD deficiency (Roe and Brunengraber, 2015). Forty-one of the participants, had been using MCT prior to the study. All infants and children also received a daily L-carnitine supplement of 100 mg/kg divided four times daily with a maximum dose of a 1,000 mg. The average frequency of serious clinical complications was reduced from ∼60% on conventional diet therapy to ∼10% with triheptanoin and carnitine treatment, and mortality decreased from ∼65% on conventional diet therapy to 3.8%. Specifically, episodes of rhabdomyolysis that required admission reduced from 85 to 31%. Reported weakness decreased from 92 to 12%. Hepatomegaly and elevated liver enzymes were present in 46% of patients prior to treatment compared to 6% while on triheptanoin. Liver abnormalities were seen during intercurrent illness but resolved rapidly with treatment. Hypoglycemia was frequent with patients on conventional diets (42%) but was not reported after initiation of triheptanoin treatment. Cardiomyopathy was identified during the first year of life in 18 of the 52 patients prior to treatment with triheptanoin, while only one patient with VLCAD deficiency developed cardiomyopathy while on triheptanoin.

Case histories of several patients reported in this publication are noteworthy. Following the neonatal death of two affected siblings, one neonate with CACT was treated with triheptanoin at 18 h of life and remained asymptomatic for the first 7 months of life. Another patient with VLCAD deficiency and a family history of an affected sibling who died of hypertrophic cardiomyopathy was enrolled at 22 days of age and remained asymptomatic at 14 years of age. A neonate with LCHAD deficiency was treated at 15 days of age and remained well for 15 years on triheptanoin and carnitine, with occasionally illness-induced episodes of rhabdomyolysis. Two pregnant females with VLCAD deficiency were included in this review. One had history of multiple episodes of rhabdomyolysis during the third trimester with her 2 previous pregnancies. She was enrolled in the study at 26 weeks of gestational age with mild hepatomegaly, muscle weakness, and decreased endurance for day to day activities. After starting triheptanoin, her muscle strength and endurance increased, while her hepatomegaly resolved. She had no episode of rhabdomyolysis and her CPK levels remained normal until the delivery of a healthy baby. The second was a 34 years old treated at 20 weeks of gestation for weakness and a history of multiple episodes of rhabdomyolysis that required hospitalization after puberty. On triheptanoin her weakness resolved, and she had no muscle pain, weakness, or episodes of rhabdomyolysis for the remainder of the pregnancy.

A retrospective, comprehensive medical record review study reviewed data from 20 (9 VLCAD, 5 LCHAD, 3 CPT II, 2 TFP, and 1 CACT deficient) of a total 24 patients with LC-FAOD who were treated for up to 12.5 years (median 8.7 years) with triheptanoin as part of a compassionate use protocol (Vockley et al., 2015). Major medical events were collected from primary medical records, including hospitalizations and emergency room admissions and home treatment of rhabdomyolysis (CPK >5,000U/L), hypoglycemia and cardiomyopathy (Vockley et al., 2015). Analyses of data of an additional 4 infants that received triheptanoin at ≤6 m old were conducted separately for events of interest, because the time period prior to initiation of triheptanoin treatment was not sufficient to allow an accurate comparison of pre and post treatment events. About 85% of the patients received MCT oil and almost all were on a low-fat high carbohydrate diet. Total hospitalizations and hospital days per year were reduced by 35 and 67%, respectively, following initiation of triheptanoin treatment. Nine patients were experiencing hypoglycemia events prior to triheptanoin treatment., and these were essentially eliminated post treatment. Although the hospitalization rate due to rhabdomyolysis did not change substantially in eleven patients, their mean hospital days/year decreased by 60% on triheptanoin treatment. Six patients with cardiomyopathy participated in the study. The cardiomyopathy resolved prior to treatment in one patient with VLCAD deficiency, the cardiac function improved in three patients (CACT, LCHAD, and VLCAD deficiencies), and one patient (VLCAD deficient) remained stable while on treatment. One patient with neonatal CPTII deficiency had extremely poor pretreatment function with cardiac fibrosis, and eventually required a heart transplant. However, this patient experienced a dramatic improvement in muscle tone and glucose control. In the four infants who began treatment with triheptanoin before 6 months of age, the median post-treatment follow up was 9.5 years, and the mean number of hospital days/year was reduced by 91%. The overall hospitalization event rate was 13.01 annualized events/year pretreatment vs. 1.36 events/year post treatment, though the number of patients was small and there was high variability in the pretreatment period.

A case series of 10 patients with LC-FAOD who presented with cardiomyopathy in infancy and childhood and received triheptanoin on a compassionate basis included 4 patients with VLCAD, two with CACT, two with TFP, and two with LCHAD deficiency (Vockley et al., 2016b). One of the patients with VLCAD deficiency was also included in the previous retrospective study (Vockley et al., 2015). Nine patients had cardiomyopathy diagnosed within the first year of life and most of the patients required ECMO and/or vasopressors during their episode of decompensation with cardiomyopathy. Ejection fraction was available for 9 patients prior to triheptanoin treatment and its mean value was 28% (range 12–45%). Following initiation of triheptanoin, patients started showing improvements in their EF between days 2–21 and their mean EF increased to 60% (range 33–71%). Eight of the patients ultimately achieved an EF within the normal range. Two patients were weaned from ECMO within 2–3 days of initiating triheptanoin. In others, ECMO and/or vasopressors was discontinued within 8–10 days following initiation of treatment. Two infants died, both at 3.5 months of age; one with VLCADD died of sepsis and one with TFP had refractory cardiogenic shock.

In a double blind randomized controlled trial, 32 subjects with LC-FAODs (11 with CPTII, 9 with VLCAD, 12 with LCHAD/TFP deficiency) older than 7 years old, received a diet containing 20% of their total energy intake from either triheptanoin (C7) or trioctanoin (C8) for 4 months (Gillingham et al., 2017). Primary outcomes included changes in total energy expenditure, cardiac function by echocardiogram, exercise tolerance, and phosphocreatine recovery following acute exercise. Secondary outcomes included body composition, blood biomarkers, and adverse events, including incidence of rhabdomyolysis. A 3-day diet record analysis during the 4-month treatment period showed that participants consumed approximately 16 and 14% of total reported energy intake from C7 and C8, respectively. All patients had a prior history of recurrent episodes of rhabdomyolysis requiring hospitalization. All patients except for one with VLCADD had normal cardiac function. A young adult with LCHAD had recently experienced sudden cardiac arrest with resuscitation. Echocardiographic data was only available for 10 subjects in the C7 and 11 subjects in the C8 group due a change in data collection part way through the study. After 4 months, patients on C7 had increased left ventricular ejection fraction (LVEF) by 7.4%, with an 8.0% decrease in LV wall mass on their resting echocardiogram. They were able to accomplish the same level of aerobic exercise at a significantly lower maximal heart rate, suggesting reverse cardiac remodeling and improved cardiorespiratory fitness. There was no significant difference in musculoskeletal symptoms between patients treated with C7 and C8; there were 7 hospitalizations for acute rhabdomyolysis in both groups, with no difference in the hospitalization length or peak CPK elevations during admissions. The other primary and secondary parameters were not different between the two groups. The main limitation of this study was the relatively short 4 months follow-up period. Thus, the clinical significance of cardiac function improvement in patients with normal ejection function could not be determined.

A single-arm, open-label, multi-center phase 2 study evaluated 29 patients (10 months–58 years old) with history of severe LC-FAOD for a total study duration of 78 weeks (Vockley et al., 2017, 2019a). Enrolled patients had history of severe LC-FAOD evidenced by ongoing musculoskeletal disease (86%), cardiomyopathy (∼7%), and hypoglycemia (10%). They received triheptanoin (UX007) in a titrating dose to 25–35% (mean 27.5%) of their total daily caloric intake, following a 4-week run in on their current treatment. All patients followed a low-fat high-carbohydrate diet prior to entering the study, 76% received carnitine supplementation, and 93% were on MCT. The objectives of the study were to evaluate changes in muscle function, exercise tolerance, and health-related quality of life following 24 weeks of treatment on triheptanoin (Vockley et al., 2017), as well as its impact on major clinical events associated with LC-FAOD over 78 weeks of treatment (Vockley et al., 2019a). An external control was employed with each patient’s results on treatment compared to the year prior to starting the study. Patients were additionally evaluated on age/condition-eligible endpoints, including 12-min walk test (12MWT), cycle ergometry and age appropriate questionnaires to assess their health-related quality of life (HR-QoL). At week 18, eight eligible patients showed a 28% increase from baseline in 12MWT distance. At week 24, seven eligible patients showed a 60% increase over baseline in watts generated through cycle ergometry, and 5 adults reported significant improvements in physical and mental component scores on the HR-QoL scale compared to baseline. There was no difference in the physical health and psychosocial summary score in 5 pediatric patients compared to baseline. Eighty six percent of participants (25/29) elected to continue treatment in the 78 weeks extension period and pediatric patients now showed a significant increase in the physical health summary scores. Overall, there was a 48% reduction in the mean annualized major event rate and a 50.3% decrease in the mean annualized duration rate on triheptanoin. The mean annualized hospitalization rate and hospitalization duration was reduced by 53.1 and 51.5%, respectively. Rhabdomyolysis was the most common major event, and the mean annualized rhabdomyolysis rate decreased by 36.1%, while the mean annualized duration rate for rhabdomyolysis was reduced by 29.3%. The mean annualized hypoglycemia event rate decreased by 92.8%. The single hypoglycemia event that occurred during UX007 treatment, did not result in hospitalization, and occurred early during the UX007 treatment. Eleven hospitalizations due to hypoglycemia were recorded prior to UX007, with 2 episodes requiring ICU admission. There was a 69.7% decrease in annualized mean cardiomyopathy events (3 events in the pretreatment period, 1 event after UX007). While the study was a small size and unblinded, the objective nature of major events was well standardized in the historic control and treatment analyses.

Adverse events in all of the clinical trials were similar with C7 compared to C8 treatment with domination by gastrointestinal symptoms and/or those related to the underlying disease. Very few patients elected to discontinue C7 treatment during the trials, and those that did, did so because of gastrointestinal symptoms.

## Use of Triheptanoin in Other Disorders

Triheptanoin has been studied as a potential therapy for several other disorders (Table 3). Most of these have significant neurologic phenotypes including seizures. Pyruvate carboxylase deficiency that results in depletion oxaloacetate, another cycle intermediate is probably the most attractive of this group. This enzyme is responsible for ensuring proper balance of oxaloacetate, the TCA cycle intermediate that together acetyl-CoA form citrate, for the TCA cycle to function normally. The anaplerotic effect of triheptanoin could in theory directly compensate for this defect; however, studies to date have reported conflicting results (Mochel et al., 2005; Breen et al., 2014). A clinical trial for treatment of seizures in GLUT1 deficiency failed to show improvement in a cohort of patients who had failed to respond to MCT oil, but a direct comparison was not performed (Mochel, 2017; Hainque et al., 2019; Hainque et al., 2020).

**TABLE 3:** Use of triheptanoin in other disorders.

Study (References)	Design	Sample size	Disease	Clinical presentation	Intervention	Outcomes
Mochel et al., 2005	Case report	1	PCD	At birth w lactic acidosis (L 17 mmol/L), ketoacidosis, elevated LFTs, hypotonia, hyperammonemia, mild hepatomegaly, bilateral periventricular pseudocysts	Triheptanoin on d7, 35% of total calories. Loading dose 2 mg/kg, then 0.5 mg/kg q3h	Lactic acidosis decreased, NH3 normalized, liver failure reversed in <48 h, resolution of pseudocysts at 3 m. At 4 m: increased lactate and ketones. Died at 6 m from metabolic decompensation due to infection
Breen et al., 2014	Case report	2	PCD	Pt 1: presented at birth with lactic acidosis (L19.5 mmol/L), ketonuria, hyperammonemia, mild liver dysfunction, brain anomalies. Pt 2: presented at birth w severe lactic acidosis (L: 32 mmol/L), hyperammonemia, brain cysts.	Triheptanoin at 11 d (up to 36.5% total calories) Triheptanoin at 3 w (32% of total calories).	No improvement in clinical and biochemical parameters. Persistent lactic acidosis. Pt 1 died at 7 m of pneumonia. Pt 2 died at 8 m of L lung collapse, severe acidosis.
Pascual et al., 2014	Open label	14 (age 2—28 y)	GLUT1-DS	Varying degrees of disease severity, not receiving KD	Triheptanoin ∼1 gr/kg/d administered for 3 m. 12/14 pts completed the study. 5/14 had MRI imaging and 9/14 completed neuropsychological testing.	All patients had decreased spike-wave seizures. Most pts showed increase in cerebral metabolic rate and improved neuropsychological performance.
Mochel et al., 2016	Open label	8 (age 7–47 y)	GLUT1-DS	Pts with chronic history of non-epileptic paroxysmal motor disorders, limb weakness, headache, drowsiness, and dysphoria. Three pts had mild cognitive deficit. None received KD	Triheptanoin 1 gr/kg/d, div TID-QID for 2 m. Two pts were non-compliant	90% reduction of non-epileptic paroxysmal manifestations that recurred w treatment withdrawal. Brain bio-energetic profile (f-MRS) normalized but returned to abnormal with treatment withdrawal
Hainque et al., 2019	Open label, extended protocol of Mochel et al. (2016)	5	GLUT1-DS	Pts with history of non-epileptic paroxysmal motor disorders, limb weakness, headache, drowsiness, and dysphoria. Three pts had mild cognitive deficit. None received KD.	Triheptanoin 1 gr/kg/d, div TID-QID for 3 years. One pt withdrew.	Triheptanoin led to dramatic (97%) and sustained reduction of paroxysmal events
Hainque et al., 2020	Open label	4	GLUT1-DS	Pts with persistent paroxysmal manifestations previously on KD	Triheptanoin was gradually introduced in about 30% of pt’s daily caloric intake. Pts were followed for 3 y	During transition from KD to triheptanoin, 3 pts had increased paroxysmal episodes. One pt quit after transition due to symptoms’ worsening. At 3 y, 2 pts had sustained reduction of total events, 1 pt had stable events.
Roe et al., 2010	Open label	5 adults	APBD	Pts with variable APBD manifestations including difficulty walking, peripheral neuropathy, poor balance, weakness, neurogenic bladder, ptosis.	Triheptanoin at 1.0–1.5 gr/kg/d div QID for 30–35% daily caloric intake for 6–8 m.	All pts perceived stabilization of disease progression, and strength increase. Most patients had 6-min walk improvement, ptosis elimination and decreased leg burning. After 6–8 m on therapy, pts sensed plateau in rate of improvement.
Calvert et al., 2018	Open-label, non-randomized, uncontrolled phase I	12 (3—18 y)	Medically refractory epilepsy	Pts with epilepsy with at least 2 motor seizures per 2-wk period over 2 m before enrollment, despite treatment with at least one AED.	Triheptanoin titrated for 3–6 w up to a max dose of 35% caloric intake or 100 ml/d, div over 3–5 doses w meals. Duration of treatment on maintenance was 12 w.	8 pts completed the study; 5/8 had >50% reduction in seizure frequency. 2/8 had initial improvement but then relapsed although seizure control was better compared to pretreatment period. 1 pt had no changes. 4 pts exited the study due to GI side effects (diarrhea, abdominal pain). Seizures returned in 3 pts that went on extension.
Borges et al., 2019	Double blind, randomized controlled trial	34 adults	Medicall y refractory epilepsy	Adult pts	Pts randomized to receive triheptanoin or MCT (17 pts to each arm) add-on treatment. Dose titrated over 3 w and maintained for another 12 w. Max dose 100 ml/d div TID	Number and type of adverse effects were similar between arms. Study not designed to compare anti-seizure effects. However, 5/11 pts on MCT showed >50% reduction of focal unaware seizures, 1/9 pts on triheptanoin showed seizure reduction.
Borges et al., 2020	Open label, extension trial	10	Medically refractory epilepsy	Adult pts with drug resistant epilepsy who had previously completed a 12 w randomized controlled trial of add-on MCT vs. triheptanoi, received add-on triheptanoin for 48 w.	Dose titrated over 3 w to a maximum tolerated dose of 100 ml/d or 35% caloric intake, then maintenance dose ×48 w	5/10 pts showed >50% reduction in seizure frequency. 2/10 completed 48 w trial. 8 pts withdrew due to lack of efficacy (3), seizure increase (1), other reasons excluding adverse effects (4).
Mochel et al., 2010	Open label, short-term	6	HD	Mild-to-moderate HD, measured by the UHDRS clinical scale.	Triheptanoin at 1 gr/kg/d for 4 d.	2 pts had end-exercise muscle acidosis that corrected 4 d post triheptanoin treatment. All pts had increased serum IGF1 after treatment. There was no significant change in UHDRS scores before and after triheptanoin.

*AED, anti-epileptic drug; APBD, adult polyglucosan body disease; d, day(s); div, divided; f-MRS, functional^31^ P-NMR spectroscopy; GLUT1-DS, glucose transporter type 1 deficiency syndrome; h, hour(s); HD, Huntington’s disease; KD, ketogenic diet; L, lactate; LFTs, liver function tests; m, month(s); MCT, medium−chain triglycerides; NH3, ammonia; PCD, pyruvate carboxylase deficiency; pt, patient; UHDRS, unified Huntington Disease Rating Scale; w, week(s); y, year(s).*

### FAO and Cardiolipin Metabolism

Cardiolipin (CL) is the main phospholipid in the inner mitochondrial membrane (IMM) (Mejia et al., 2014; Ren et al., 2014). Unlike other phospholipids, it has 4 fatty acyl chains. In the heart and muscle, its predominant form is tetralinoleoylcardiolipin (L4CL); which has 4 symmetrical linoleoyl chains giving it a coned shape. This unique molecular structure and location is critical to maintain the integrity of the cristae of the IMM, cristae, and respiratory chain complexes (OXPHOS) to optimize energy production capacity as demonstrated by the severe cardiac manifestations seen in Barth syndrome due to *TAZ* gene mutations. This defect alters the composition and distribution of cardiolipin species leading to a major reduction in L4CL species, and accumulation of an asymmetrical form of monolysocardiolipin, leading to disorganization and destabilization of the IMM and OXPHOS supercomplexes, reduced ATP production and cardiac dysfunction (Ikon and Ryan, 2017). Recently, it has been demonstrated that the MTPα/HADHA subunit also encompasses a monolysocardiolipin acetyltransferase (MLCLAT) activity critical to cardiolipin remodeling (Taylor et al., 2012; Miklas et al., 2019). MLCLAT is a portion of the C-terminus (59 kDa) of MTPα/HADHA (75 kDa) minus the final 227 amino acids. MLCLAT has *in vitro* specificity for linoleoyl-CoA > oleoyl-CoA > palmitoyl-CoA, and specificity for acylation of monolysocardiolipin. Pluripotential stem cells made from an HADAH deficient patient differentiated to a cardiomyocyte phenotype show impaired calcium metabolism and repolarization kinetics and mitochondrial bioenergetics with abnormal mitochondrial structure and maturation of cardiolipin (Miklas et al., 2019). The mitochondrial proton leak improved with treatment with SS-31, a cardiolipin binding peptide that improves mitochondrial stability, emphasizing the role of LCHAD in cardiolipin metabolism. Some symptoms seen in LCHAD/TFP deficient patients are unique among the LC-FAODs (retinopathy and peripheral neuropathy) and are more in keeping with those seen in mitochondrial myopathies. Thus the role of CL abnormalities related to the development of these symptoms in LCHAD/TFP deficiency remains to be determined.

## Summary

In summary, patients with LC-FAODs have a secondary deficit of TCA cycle intermediates that further impairs the primary energy deficit due to defective FAD. Triheptanoin is an anaplerotic medium chain triglyceride that provides both even and odd chain substrates to the TCA cycle, restoring its balance and improving ATP generation in experimental systems. LC-FAOD Patients treated with triheptanoin show significant improvement compared to pretreatment, with the greatest improvements in glucose homeostasis and cardiomyopathy. While episodes of rhabdomyolysis decreased significantly with triheptanoin treatment, the effect was less than with the other symptoms, suggesting a role for other pathophysiologic mechanisms and demonstrating the need for additional therapies. The use of triheptanoin in a variety of other disorders remains under investigation.

## Author Contributions

ES performed the literature review and wrote the first draft of the manuscript. AA performed metabolomic experiments. SD and A-WM helped supervise metabolomic experiments and edited manuscript. JV supervised entire project, edited manuscript, and made figures. All authors contributed to the article and approved the submitted version.

## Conflict of Interest

The authors declare that the research was conducted in the absence of any commercial or financial relationships that could be construed as a potential conflict of interest.

## Footnotes

1 https://www.metabolon.com

## References

[B1] Baruteau J. Sachs P. Broué P. Brivet M. Abdoul H. Vianey-Saban C. (2013). Clinical and biological features at diagnosis in mitochondrial fatty acid beta-oxidation defects: a French pediatric study of 187 patients. *J. Inherit. Metab. Dis.* 36 795–803. 10.1007/s10545-012-9542-6 23053472

[B2] Borges K. Kaul N. Germaine J. Carrasco-Pozo C. Kwan P. O’Brien T. J. (2020). Open-label long-term treatment of add-on triheptanoin in adults with drug-resistant epilepsy. *Epilepsia Open* 5 230–239. 10.1002/epi4.12391 32524048 PMC7278596

[B3] Borges K. Kaul N. Germaine J. Kwan P. O’Brien T. J. (2019). Randomized trial of add-on triheptanoin vs medium chain triglycerides in adults with refractory epilepsy. *Epilepsia Open* 4 153–163. 10.1002/epi4.12308 30868125 PMC6398112

[B4] Breen C. White F. J. Scott C. A. Heptinstall L. Walter J. H. Jones S. A. (2014). Unsuccessful treatment of severe pyruvate carboxylase deficiency with triheptanoin. *Eur. J. Pediatr.* 173 361–366. 10.1007/s00431-013-2166-5 24114256

[B5] Calvert S. Barwick K. Par M. Ni Tan K. Borges K. (2018). A pilot study of add-on oral triheptanoin treatment for children with medically refractory epilepsy. *Eur. J. Paediatr. Neurol.* 22 1074–1080. 10.1016/j.ejpn.2018.07.014 30126760

[B6] Dehaven C. D. Evans A. M. Dai H. Lawton K. A. (2010). Organization of GC/MS and LC/MS metabolomics data into chemical libraries. *J. Cheminform.* 2:9. 10.1186/1758-2946-2-9 PMC2984397 20955607

[B7] Deng S. Zhang G. F. Kasumov T. Roe C. R. Brunengraber H. (2009). Interrelations between C4 ketogenesis, C5 ketogenesis, and anaplerosis in the perfused rat liver. *J. Biol. Chem.* 284 27799–27807. 10.1074/jbc.m109.048744 19666922 PMC2788830

[B8] Dobrowolski S. F. Alodaib A. Karunanidhi A. Basu S. Holecko M. Lichter-Konecki U. (2020). Clinical, biochemical, mitochondrial, and metabolomic aspects of methylmalonate semialdehyde dehydrogenase deficiency: report of a fifth case. *Mol. Genet. Metab.* 129 272–277. 10.1016/j.ymgme.2020.01.005 32151545

[B9] Evans A. M. DeHaven C. D. Barrett T. Mitchell M. Milgram E. (2009). Integrated, nontargeted ultrahigh performance liquid chromatography/electrospray ionization tandem mass spectrometry platform for the identification and relative quantification of the small-molecule complement of biological systems. *Anal. Chem.* 81 6656–6667. 10.1021/ac901536h 19624122

[B10] Gillingham M. B. Heitner S. B. Martin J. Rose S. Goldstein A. El-Gharbawy A. H. (2017). Triheptanoin versus trioctanoin for long-chain fatty acid oxidation disorders: a double blinded, randomized controlled trial. *J. Inherit. Metab. Dis.* 40 831–843. 10.1007/s10545-017-0085-8 28871440 PMC6545116

[B11] Gu L. Zhang G. F. Kombu R. S. Allen F. Kutz G. Brewer W. U. (2010). Parenteral and enteral metabolism of anaplerotic triheptanoin in normal rats. II. Effects on lipolysis, glucose production, and liver acyl-CoA profile. *Am. J. Physiol. Endocrinol. Metab.* 298 E362–E371. 19903863 10.1152/ajpendo.00384.2009 PMC2822475

[B12] Hainque E. Gras D. Meneret A. Atencio M. Luton M. P. Barbier M. (2019). Long-term follow-up in an open-label trial of triheptanoin in GLUT1 deficiency syndrome: a sustained dramatic effect. *J. Neurol. Neurosurg. Psychiatry* 90 1291–1293. 10.1136/jnnp-2018-320283 30948626 PMC6860903

[B13] Hainque E. Meneret A. Gras D. Atencio M. Luton M. P. Barbier M. (2020). Transition from ketogenic diet to triheptanoin in patients with GLUT1 deficiency syndrome. *J. Neurol. Neurosurg. Psychiatry* 91 444–445. 10.1136/jnnp-2019-321694 31694879

[B14] Ikon N. Ryan R. O. (2017). Barth syndrome: connecting cardiolipin to cardiomyopathy. *Lipids* 52 99–108. 10.1007/s11745-016-4229-7 28070695 PMC5288132

[B15] Kinman R. P. Kasumov T. Jobbins K. A. Thomas K. R. Adams J. E. Brunengraber L. N. (2006). Parenteral and enteral metabolism of anaplerotic triheptanoin in normal rats. *Am. J. Physiol. Endocrinol. Metab.* 291 E860–E866. 16705058 10.1152/ajpendo.00366.2005

[B16] Knottnerus S. J. G. Bleeker J. C. Wüst R. C. I. Ferdinandusse S. IJlst L. Wijburg F. A. (2018). Disorders of mitochondrial long-chain fatty acid oxidation and the carnitine shuttle. *Rev. Endocr. Metab. Disord.* 19 93–106. 10.1007/s11154-018-9448-1 29926323 PMC6208583

[B17] Liang K. Li N. Wang X. Dai J. Liu P. Wang C. (2018). Cryo-EM structure of human mitochondrial trifunctional protein. *Proc. Natl. Acad. Sci. U.S.A.* 115 7039–7044. 10.1073/pnas.1801252115 29915090 PMC6142257

[B18] Lindner M. Hoffmann G. F. Matern D. (2010). Newborn screening for disorders of fatty-acid oxidation: experience and recommendations from an expert meeting. *J. Inherit. Metab. Dis.* 33 521–526. 10.1007/s10545-010-9076-8 20373143

[B19] Longo N. (2016). Primary carnitine deficiency and newborn screening for disorders of the carnitine cycle. *Ann. Nutr. Metab.* 68(Suppl. 3) 5–9. 10.1159/000448321 27931018

[B20] Marsden D. Bedrosian C. Vockley J. (2020). Impact of newborn screening on the reported incidence and clinical outcomes associated with medium- and long-chain fatty acid oxidation disorders. *Genet. Med.* 10.1038/s41436-020-01070-0 PMC8105167 33495527

[B21] Mejia E. M. Nguyen H. Hatch G. M. (2014). Mammalian cardiolipin biosynthesis. *Chem. Phys. Lipids* 179 11–16. 10.1016/j.chemphyslip.2013.10.001 24144810

[B22] Miklas J. W. Clark E. Levy S. Detraux D. Leonard A. Beussman K. (2019). TFPa/HADHA is required for fatty acid beta-oxidation and cardiolipin re-modeling in human cardiomyocytes. *Nat. Commun.* 10:4671. 10.1038/s41467-019-12482-1 PMC6789043 31604922

[B23] Mochel F. (2017). Triheptanoin for the treatment of brain energy deficit: a 14-year experience. *J. Neurosci. Res.* 95 2236–2243. 10.1002/jnr.24111 28688166

[B24] Mochel F. DeLonlay P. Touati G. Brunengraber H. Kinman R. P. Rabier D. (2005). Pyruvate carboxylase deficiency: clinical and biochemical response to anaplerotic diet therapy. *Mol. Genet. Metab.* 84 305–312. 10.1016/j.ymgme.2004.09.007 15781190

[B25] Mochel F. Duteil S. Marelli C. Jauffret C. Barles A. Holm J. (2010). Dietary anaplerotic therapy improves peripheral tissue energy metabolism in patients with Huntington’s disease. *Eur. J. Hum. Genet.* 18 1057–1060. 10.1038/ejhg.2010.72 20512158 PMC2987415

[B26] Mochel F. Hainque E. Gras D. Adanyeguh I. M. Caillet S. Héron B. (2016). Triheptanoin dramatically reduces paroxysmal motor disorder in patients with GLUT1 deficiency. *J. Neurol. Neurosurg. Psychiatry* 87 550–553. 10.1136/jnnp-2015-311475 26536893 PMC4853553

[B27] Nahabet E. H. Gatherwright J. Vockley J. Henderson N. Tomei K. L. Grigorian A. P. (2016). Postnatal pancraniosynostosis in a patient with infantile hypophosphatasia. *Cleft Palate Craniofac. J.* 53 741–744. 10.1597/15-027 26171568

[B28] Pascual J. M. Liu P. Mao D. Kelly D. I. Hernandez A. Sheng M. (2014). Triheptanoin for glucose transporter type I deficiency (G1D): modulation of human ictogenesis, cerebral metabolic rate, and cognitive indices by a food supplement. *JAMA Neurol.* 71 1255–1265. 10.1001/jamaneurol.2014.1584 25110966 PMC4376124

[B29] Quell J. D. Römisch-Margl W. Colombo M. Krumsiek J. Evans A. M. Mohney R. (2017). Automated pathway and reaction prediction facilitates in silico identification of unknown metabolites in human cohort studies. *J. Chromatogr. B Analyt. Technol. Biomed. Life Sci.* 1071 58–67. 10.1016/j.jchromb.2017.04.002 28479069

[B30] Rector R. S. Payne R. M. Ibdah J. A. (2008). Mitochondrial trifunctional protein defects: clinical implications and therapeutic approaches. *Adv. Drug. Deliv. Rev.* 60 1488–1496. 10.1016/j.addr.2008.04.014 18652860 PMC2848452

[B31] Ren M. Phoon C. K. Schlame M. (2014). Metabolism and function of mitochondrial cardiolipin. *Prog. Lipids Res.* 55 1–16. 10.1016/j.plipres.2014.04.001 24769127

[B32] Roe C. R. Bottiglieri T. Wallace M. Arning E. Martin A. (2010). Adult polyglucosan body disease (APBD): anaplerotic diet therapy (Triheptanoin) and demonstration of defective methylation pathways. *Mol. Genet. Metab.* 101 246–252. 10.1016/j.ymgme.2010.06.017 20655781

[B33] Roe C. R. Brunengraber H. (2015). Anaplerotic treatment of long-chain fat oxidation disorders with triheptanoin: review of 15 years experience. *Mol. Genet. Metab.* 116 260–268. 10.1016/j.ymgme.2015.10.005 26547562 PMC4712637

[B34] Roe C. R. Sweetman L. Roe D. S. David F. Brunengraber H. (2002). Treatment of cardiomyopathy and rhabdomyolysis in long-chain fat oxidation disorders using an anaplerotic odd-chain triglyceride. *J. Clin. Invest.* 110 259–269. 10.1172/jci0215311 12122118 PMC151060

[B35] Roe C. R. Yang B. Z. Brunengraber H. Roe D. S. Wallace M. Garritson B. K. (2008). Carnitine palmitoyltransferase II deficiency: successful anaplerotic diet therapy. *Neurology* 71 260–264. 10.1212/01.wnl.0000318283.42961.e9 18645163 PMC2676979

[B36] Spiekerkoetter U. Bennett M. J. Ben-Zeev B. Strauss A. W. Tein I. (2004). Peripheral neuropathy, episodic myoglobinuria, and respiratory failure in deficiency of the mitochondrial trifunctional protein. *Muscle Nerve* 29 66–72. 10.1002/mus.10500 14694500

[B37] Spiekerkoetter U. Lindner M. Santer R. Grotzke M. Baumgartner M. R. Boehles H. (2009b). Treatment recommendations in long-chain fatty acid oxidation defects: consensus from a workshop. *J. Inherit. Metab. Dis.* 32 498–505. 10.1007/s10545-009-1126-8 19452263

[B38] Spiekerkoetter U. Lindner M. Santer R. Grotzke M. Baumgartner M. R. Boehles H. (2009a). Management and outcome in 75 individuals with long-chain fatty acid oxidation defects: results from a workshop. *J. Inherit. Metab. Dis.* 32 488–497. 10.1007/s10545-009-1125-9 19399638

[B39] Spiekerkoetter U. Mayatepek E. (2010). Update on mitochondrial fatty acid oxidation disorders. *J. Inherit. Metab. Dis.* 33 467–468. 10.1007/s10545-010-9208-1 20842433

[B40] Swigonova Z. Mohsen A. W. Vockley J. (2009). Acyl-CoA dehydrogenases: dynamic history of protein family evolution. *J. Mol. Evol.* 69 176–193. 10.1007/s00239-009-9263-0 19639238 PMC4136416

[B41] Taylor W. A. Mejia E. M. Mitchell R. W. Choy PC. Sparagna GC. Hatch GM. (2012). Human trifunctional protein alpha links cardiolipin remodeling to beta-oxidation. *PLoS One* 7:e48628. 10.1371/journal.pone.0048628 23152787 PMC3494688

[B42] Tucci S. (2017). Very long-chain acyl-CoA dehydrogenase (VLCAD-) deficiency-studies on treatment effects and long-term outcomes in mouse models. *J. Inherit. Metab. Dis.* 40 317–323. 10.1007/s10545-017-0016-8 28247148

[B43] Tucci S. Behringer S. Spiekerkoetter U. (2015). De novo fatty acid biosynthesis and elongation in very long-chain acyl-CoA dehydrogenase-deficient mice supplemented with odd or even medium-chain fatty acids. *FEBS J.* 282 4242–4253. 10.1111/febs.13418 26284828

[B44] Tucci S. Floegel U. Beermann F. Behringer S. Spiekerkoetter U. (2017). Triheptanoin: long-term effects in the very long-chain acyl-CoA dehydrogenase-deficient mouse. *J. Lipid Res.* 58 196–207. 10.1194/jlr.m072033 27884962 PMC5234721

[B45] Vieira de Melo I. S. Da Rocha Ataide T. Lima de Oliveira S. Bezerra Bueno N. Duarte de Freitas J. Goulart Sant’Ana A. E. (2015). Hepatic fatty acid profile of rats fed a triheptanoin-based ketogenic diet. *Nutr. Hosp.* 32 265–269. 26262726 10.3305/nh.2015.32.1.9033

[B46] Vockley J. Bennett M. J. Gillingham M. B. (2016a). “Mitochondrial fatty acid oxidation disorders,” in *The Online Metabolic and Molecular Bases of Inherited Disease*, eds Beaudet A. L. Vogelstein B. Kinzler K. W. Antonarakis S. E. Ballabio A. Gibson K. M. Mitchell G. (New York, NY: The McGraw-Hill Companies, Inc).

[B47] Vockley J. Burton B. Berry G. T. Longo N. Phillips J. Sanchez-Valle A. (2017). UX007 for the treatment of long chain-fatty acid oxidation disorders: safety and efficacy in children and adults following 24weeks of treatment. *Mol. Genet. Metab.* 120 370–377. 10.1016/j.ymgme.2017.02.005 28189603

[B48] Vockley J. Burton B. Berry G. T. Longo N. Phillips J. Sanchez-Valle A. (2019a). Results from a 78-week, single-arm, open-label phase 2 study to evaluate UX007 in pediatric and adult patients with severe long-chain fatty acid oxidation disorders (LC-FAOD). *J. Inherit. Metab. Dis.* 42 169–177. 10.1002/jimd.12038 30740733 PMC6348052

[B49] Vockley J. Charrow J. Ganesh J. Eswara M. Diaz G. A. McCracken E. (2016b). Triheptanoin treatment in patients with pediatric cardiomyopathy associated with long chain-fatty acid oxidation disorders. *Mol. Genet. Metab.* 119 223–231. 10.1016/j.ymgme.2016.08.008 27590926 PMC5083220

[B50] Vockley J. Dobrowolski S. F. Arnold G. L. Guerrero R. B. Derks T. G. J. Weinstein D. A. (2019b). Complex patterns of inheritance, including synergistic heterozygosity, in inborn errors of metabolism: Implications for precision medicine driven diagnosis and treatment. *Mol. Genet. Metab.* 128 1–9. 10.1016/j.ymgme.2019.07.011 31358473 PMC8931500

[B51] Vockley J. Marsden D. McCracken E. DeWard S. Barone A. Hsu K. (2015). Long-term major clinical outcomes in patients with long chain fatty acid oxidation disorders before and after transition to triheptanoin treatment–A retrospective chart review. *Mol. Genet. Metab.* 116 53–60. 10.1016/j.ymgme.2015.06.006 26116311 PMC4561603

[B52] Vockley J. Singh R. H. Whiteman D. A. (2002). Diagnosis and management of defects of mitochondrial beta-oxidation. *Curr. Opin. Clin. Nutr. Metab. Care* 5 601–609. 10.1097/00075197-200211000-00002 12394635

[B53] Wilcken B. Haas M. Joy P. Wiley V. Bowling F. Carpenter K. (2009). Expanded newborn screening: outcome in screened and unscreened patients at age 6 years. *Pediatrics* 124 e241–e248. 19620191 10.1542/peds.2008-0586

[B54] Xia C. Fu Z. Battaile K. P. Kim J. P. (2019). Crystal structure of human mitochondrial trifunctional protein, a fatty acid beta-oxidation metabolon. *Proc. Natl. Acad. Sci. U.S.A.* 116 6069–6074. 10.1073/pnas.1816317116 30850536 PMC6442613

